# The biospheric emergency calls for scientists to change tactics

**DOI:** 10.7554/eLife.83292

**Published:** 2022-11-07

**Authors:** Fernando Racimo, Elia Valentini, Gaston Rijo De León, Teresa L Santos, Anna Norberg, Lane M Atmore, Myranda Murray, Sanja M Hakala, Frederik Appel Olsen, Charlie J Gardner, Julia B Halder

**Affiliations:** 1 University of Copenhagen Copenhagen Denmark; 2 Scientist Rebellion Denmark Copenhagen Denmark; 3 University of Essex Colchester United Kingdom; 4 Scientist Rebellion Italy Rome Italy; 5 Scientist Rebellion UK Colchester United Kingdom; 6 Institut Pasteur Paris France; 7 Scientist Rebellion France Paris France; 8 Universidade de Lisboa Lisbon Portugal; 9 Scientist Rebellion Portugal Lisboa Portugal; 10 Norwegian University of Science and Technology Trondheim Norway; 11 Scientist Rebellion Norway Trondheim Norway; 12 University of Oslo Oslo Norway; 13 Scientist Rebellion Turtle Island Turtle Island United States; 14 University of Fribourg Fribourg Switzerland; 15 Scientist Rebellion Switzerland Fribourg Switzerland; 16 University of Copenhagen Copenhagen Denmark; 17 University of Kent Canterbury United Kingdom; 18 Scientist Rebellion UK Canterbury United Kingdom; 19 Imperial College London United Kingdom; 20 Scientist Rebellion UK London United Kingdom; eLife United Kingdom; eLife United Kingdom

**Keywords:** climate breakdown, ecology, public health, activism, civil resistance, biodiversity crisis, Human

## Abstract

Our current economic and political structures have an increasingly devastating impact on the Earth’s climate and ecosystems: we are facing a biospheric emergency, with catastrophic consequences for both humans and the natural world on which we depend. Life scientists – including biologists, medical scientists, psychologists and public health experts – have had a crucial role in documenting the impacts of this emergency, but they have failed to drive governments to take action in order to prevent the situation from getting worse. Here we, as members of the movement Scientist Rebellion, call on life scientists to re-embrace advocacy and activism – which were once hallmarks of academia – in order to highlight the urgency and necessity of systemic change across our societies. We particularly emphasise the need for scientists to engage in nonviolent civil resistance, a form of public engagement which has proven to be highly effective in social struggles throughout history.

## Introduction

We are running out of time. Ecosystems across the planet are being destroyed at an accelerating rate. The life sciences – once a field dedicated to the study of living systems and our interactions with them – are increasingly becoming sciences of the dead. Up to one million species are currently threatened with extinction, many of them within decades. This includes as many as 10% of insect species (IPBES, 2019), as well as two in five plant species (Nic Lughadha et al., 2020). Moreover, many species that are not immediately threatened by extinction are suffering population declines (Wagner, 2020). Through a combination of unfettered changes in land use, exploitative farming practices, overfishing, and fossil fuel emissions, our planet is experiencing an extinction process of unprecedented speed (WWF, 2020).

Current projections paint a grim picture for what our planet will look like in the near future, as biodiversity loss is further compounded by climate breakdown (IPBES, 2019). The Secretary-General of the United Nations, António Guterres, recently stressed that “the evidence is irrefutable: greenhouse gas emissions from fossil fuel burning and deforestation are choking our planet” (United Nations, 2021). The latest report from the Intergovernmental Panel on Climate Change (IPCC) asserts global warming is on track to exceed 2°C during this century (IPCC, 2021), and more recent estimates are even higher (Climate Action Tracker, 2022). Furthermore, research indicates that the human climate niche may dramatically shrink over the next fifty years, making large swaths of the planet incompatible with human survival, and thus forcing mass displacements of hundreds of millions of people (Xu et al., 2020). It has been estimated that the disruption of our climate system will cause around five million excess deaths annually (Bressler, 2021; Zhao et al., 2021), via massively increased risks of heat stress, droughts, crop collapses, outbreaks of diseases and other natural disasters (WHO, 2021). The effect of these climate and ecological crises is exacerbated in lower-income countries, where the capacity for response is substantially decreased due to centuries of colonial appropriation and resource depletion (Fanning et al., 2022; Hickel et al., 2022).

Life scientists have documented the extermination of species and the destruction of ecosystems in excruciating detail (Hodgson et al., 2018; Cordier et al., 2021; Schmolke et al., 2010; Fitzpatrick and Keller, 2015). Yet our measurements, predictions and conclusions are overwhelmingly ignored by politicians, who have the power to stop this process. Even as researchers write countless warnings and reports on the biospheric emergency, the situation gets worse every year (Ripple et al., 2020; Ripple et al., 2017; Pyšek et al., 2020; Georgian et al., 2022; Cavicchioli et al., 2019; Albert et al., 2021). The life sciences thus seem powerless to stop the destruction of their own subject of study.

What has gone wrong? In this article, we highlight how life scientists are failing in our duty to effectively engage with society about the biospheric emergency. We emphasise how a focus on behaviours that maintain the status quo has cornered us into accepting practices and modes of communication that run counter to our own scientific recommendations. This, in turn, is hindering the transformative societal change that is needed to avert the worst consequences of the climate and ecological crises. Motivated by an academic environment that disincentivizes (and even sanctions) social and political critique, many life scientists have resigned themselves to reporting on the consequences of the biospheric emergency, while shying away from its underlying social, economic and political causes.

We, as members of the movement Scientist Rebellion, call on life scientists to address these challenges by rediscovering forms of advocacy and activism that were a distinctive feature of previous generations of academics. To help this effort, we describe how advocacy and activism can be integrated into our scholarly responsibilities – including outreach, teaching and research – without compromising our professional integrity.

## Acknowledging failure

Life scientists study the natural world: its past, its present, its future, and its ongoing interactions with human society. In addition to producing knowledge, many scientists feel a duty to relay this knowledge to the public (Douglas, 2009). This duty is also fundamental to many mission-driven professions within the life sciences: the role of conservation biologists and medical researchers, for example, includes an explicit obligation to create and disseminate knowledge in order to preserve life (Meine et al., 2006; Romanello et al., 2021; Bennett et al., 2020).

Thus, for both personal and professional reasons, many life scientists have been trying to communicate the urgency of the climate and ecological crises for decades, so as to trigger the cultural and political mechanisms able to prevent further degradation and collapse of ecosystems worldwide (Díaz et al., 2019; Ripple et al., 2017). In its latest report, the Intergovernmental Science-Policy Platform on Biodiversity and Ecosystem Services (IPBES) calls for “fundamental, system-wide reorganisation across technological, economic and social factors, including paradigms, goals and values” (IPBES, 2019), while the IPCC urges “fundamental changes to how society functions, including changes to underlying values, worldviews, ideologies, social structures, political and economic systems, and power relationships" (IPCC, 2022c).

Yet, our societies have not developed the radical, collective and co-ordinated systems change that a planetary-scale emergency would require. Given the level of urgency, it is critical to ask how effective scientific efforts have been at producing such change and/or at galvanising politicians to take action. Here we discuss five areas where we think life scientists have failed in this regard: biodiversity conservation; food security; global public health; mental health; and life sciences education and dissemination. Each area has garnered enormous attention from life scientists, yet the situation in each gets dramatically worse every year.

### Biodiversity and conservation

Nearly one out of every eight species is threatened with extermination, and many more have dramatically declining populations (IPBES, 2019). This issue has touched virtually all research in ecology, which is increasingly morphing into a science of extinction and risk assessment (Gardner and Bullock, 2021). The biodiversity crisis garnered public attention in the 1960s with the publication of *Silent Spring* by Rachel Carson (Carson, 1962). The following decades saw some success stories, such as the Endangered Species Act in the US and the global ban on whaling, largely as a consequence of activism (Raymond, 2018; Mackenzie, 2018). Yet, these efforts were far from enough, and the preservation of biodiversity is currently at the bottom of the list of political concerns (Bradshaw et al., 2021).

The amount of funding dedicated to conservation reflects this stark reality; the spending on global protected areas is estimated to be approximately equal to the total money spent on beard-grooming products around the world (Gardner and Bullock, 2021; Waldron et al., 2020; Thomas and Deshmukh, 2019). Out of the 20 global targets agreed in 2010 as part of the Convention on Biological Diversity, zero were met by 2020 (Convention on Biological Diversity, 2020). This is despite repeated calls from researchers for increased environmental protections and the creation of research and policy organisations such as the IPBES and the International Union for the Conservation of Nature. Research papers, media opinion pieces and conference reports are clearly not creating the impact needed to stop (or even slow down) species loss and population declines (Gardner et al., 2021; Green, 2021).

### Food security

The climate and ecological crises are deeply linked to threats to food security, as the life-support systems on which humans are dependent are jointly disrupted by ecosystem decimation and climate breakdown (FAO, 2019). Out of the world’s agricultural land, 77% is used to support livestock, which is not only one of the largest sources of greenhouse gas emissions (Rojas-Downing et al., 2017; IATP, GRAIN, Heinrich Böll Stiftung, 2017) and global habitat destruction (Mundy, 2021), but also makes up only 18% of the world’s caloric supply, predominantly in high-income nations (FAO, 2022). In addition, humans are now massively reliant on a select set of commodity crops, produced via industrial-scale monoculture farming and traded by a few corporations through international markets: maize, rice and wheat provide approximately 40% of humanity’s chemical energy. They are predominantly grown in unevenly distributed continental grassland areas, which are particularly vulnerable to climate breakdown and consequent water scarcity (Dai, 2013; Smaje, 2020). Rising temperatures are directly reducing crop growth duration, effectively reducing yield (Zhao et al., 2017). Our crop production also relies on insect pollination, natural pest control and nutrient recycling by arthropods, all of which are threatened by the ongoing collapse of invertebrate populations (Wagner, 2020). These issues have been exhaustively documented (Arora, 2019; Hatfield and Prueger, 2015; Gourdji et al., 2013) and have been the topic of discussion at large international organisations (FAO, 2019).

After all this reporting and debate, are governments working to secure resilient food systems and decrease the risk of crop failures for the years ahead? The evidence points squarely against this. The rate of fossil fuel emissions has never been higher, taking us rapidly away from the planetary safe zone in which our agricultural systems have existed for millennia (IPCC, 2021). A number of studies predict declines in global crop yields of between 20% and 90% within just a few decades, as a consequence of climate change, soil erosion and the decline in key pollinator populations (Schlenker and Roberts, 2009; Arora, 2019; Hatfield and Prueger, 2015; Gourdji et al., 2013). Meanwhile, governments are neglecting to develop resilient and equitable food production and distribution strategies at the scale needed to prevent simultaneous crop collapses and famine (Tigchelaar et al., 2018; Gaupp et al., 2019). For example, a recent study showed that much of the research funded by the US Department of Agriculture is completely unrelated to sustainable agriculture, instead focusing on (unsustainable) animal farming or enhancing monoculture technologies: projects with a focus on agroecology and socioeconomic sustainability constitute just between 5% and 10% of allocated public funds (DeLonge et al., 2016).

### Global public health

The biospheric emergency is linked to the deterioration of human health, through increasing exposure to extreme climate events (Romanello et al., 2021; Vicedo-Cabrera et al., 2021; Gasparrini et al., 2015), and our decreasing ability to effectively respond to epidemics (WHO, 2021). As governments and corporations are demolishing the biosphere, they are forcing species to coexist in new ways, including new interactions between pathogens and potential hosts (Schmeller et al., 2020; Carlson et al., 2022). More than half of human pathogenic diseases are predicted to be aggravated by climate change. Emerging epidemics in livestock and crops, as well as direct effects of climate change on food production, will also increase the risk of malnutrition and famine (Romanello et al., 2021; Mora et al., 2022); undernutrition and disease will thus be overlapping threats to an increasingly vulnerable number of people worldwide.

In light of this knowledge, is the world getting ready for the impact that the biospheric emergency will have on global public health? The answer appears to be “no”. Recent studies have highlighted a remarkable lack of preparedness for future pandemics (Gibb et al., 2020; Romanello et al., 2021), with the last author on one of these studies (Kate Jones of UCL) painting a bleak picture: “We’ve been warning about this for decades. Nobody paid any attention” (Tollefson, 2020). Moreover, it is unusual for research programmes in public health and medicine to consider the potential impact of rising temperatures and extreme weather on public health. Similarly, policymakers are neglecting to account for premature deaths related to heat stress (Gasparrini et al., 2015), which has already claimed thousands of victims in 2022 (Coi and Weise, 2022).

### Mental health

There are also growing concerns about the impact of the climate and ecological crises on mental health (Hayward and Ayeb-Karlsson, 2021; Kelman et al., 2021; Royal College of Psychiatrists, 2021). A recent IPCC report found that climate change has already had a negative impact on mental health around the world, and this is expected to get worse (IPCC, 2022a). To illustrate this, we performed a Web of Science search for research articles on the topics of “eco-anxiety,” “climate anxiety” and “ecological grief”, and found that these have skyrocketed in recent years: no articles were found for any year before 2010 and there were fewer than two articles per year between 2010 and 2017. However, there were 49 such articles in 2021, and as of 13 September the figure for 2022 was 43. Indeed, despite some taxonomic confusion (Clayton, 2020), studies suggest an increasing range of negative emotions are associated with awareness of climate change (Brosch, 2021). In the US, for example, a poll found that almost half of the adults aged 18–34 reported that the stress they feel about climate change affects their daily lives (APA, 2020). More frequent exposure to intense heat is also contributing to increased aggressivity and suicide rates (Miles-Novelo and Anderson, 2019; Thompson et al., 2018).

Meanwhile, there is an ongoing shortfall in global mental health investment (WHO, 2020), and calls to action have been made for professionals to urgently address these issues (Cunsolo and Ellis, 2018; APA, 2020). Even as numerous research studies are published on the topic, funding for mitigation and adaptation strategies in this area is still nowhere near the scale needed (Berrang-Ford et al., 2021; Hayes et al., 2018).

### Life science education and dissemination

To top things off, universities do little to teach about the impacts that the climate and ecological crises will have on these subjects, and on the careers of the students who study them. Students are rarely taught about scientific advocacy or the relationship between scientific behaviours and social change (Gardner et al., 2021; Green, 2021; Leal Filho et al., 2021; Steinberger, 2022). Instead, universities have largely emphasised responding to these crises via changes in individual behaviour rather than collective action and institutional accountability (Wynes and Nicholas, 2017; Stevenson et al., 2017; Robottom and Hart, 1995). Indeed, as higher education institutions have adopted corporate structures and goals (Kleinman and Vallas, 2001), they have shifted the blame to individuals, and disincentivized modes of collective action and organising. As a result, both staff and students are left feeling hopeless and defeated at a time when, in fact, systemic change is entirely within our grasp (Stoddard et al., 2021).

### The consequences of the failure of scientific engagement

Whether we talk about threats to food security, global health or biodiversity, scientific messaging is either not getting through to the public (de Bruin et al., 2021), or is being drowned out by sophisticated misinformation campaigns (Lewandowsky, 2020). There are now entire think tanks dedicated to occluding or misinterpreting scientific findings related to the biospheric emergency (Dunlap and Jacques, 2013; Jacques et al., 2008; Lamb et al., 2020; Lewandowsky, 2021), and recent research highlights that accurate information about climate and ecology can easily be eroded by misinformation (Nyhan et al., 2022). To make matters worse, scientific responses to misinformation campaigns tend to be dispassionate and directed at those in power (who are keen to maintain the status quo), rather than passionate and directed at the people being misinformed: in short, a recipe for disaster (Steinberger, 2019).

## Changing tactics

It is clear that the current situation calls for a radical change in the way we engage with society about the biospheric emergency. What would such a change involve? Many scientists are now outlining a path forward which entails embracing advocacy and activism in academia (Gardner and Wordley, 2019; Gardner et al., 2021; Capstick et al., 2022). Advocacy involves taking a specific public stance in support of a cause – in this case, climate and ecological action (Jickling, 2003; Nelson and Vucetich, 2009). Activism refers to particularly direct forms of advocacy, such as petitioning or protesting. One form of activism that has proven to be highly effective at garnering public support, especially when done peacefully and en masse, is civil resistance (Chenoweth et al., 2011). This involves publicly confronting or refusing to obey a particular law, order, power or rule deemed to be unjust or unethical, with the potential risk for detention or arrest. By placing oneself in a position of vulnerability with respect to a ruling regime (for example, by blocking a road or occupying a building), one can generate public concern and sympathy, in ways other forms of engagement often fail to do (Thackeray et al., 2020). Indeed, civil resistance has been a major force behind societal changes over the past two centuries, including decolonisation processes, labour rights, women’s voting rights, and civil rights for people of colour (Roberts and Ash, 2009; Chenoweth, 2021).

Advocacy and activism need not be seen as a departure from our professional duties as academics, but rather as a natural adaptation of them to times of crisis (Gardner et al., 2021). Scientific freedom is indeed yoked together with responsibility (Douglas, 2021). We are in a privileged position to be able to study life in its various forms, and produce knowledge about it – intimately attached to this freedom is the responsibility to use that knowledge to protect life. As stated in *On Being A Scientist*, a handbook published by the National Academies for Science, Engineering and Medicine in the United States: “researchers also have the right to express their convictions and work for social change, and these activities need not undercut a rigorous commitment to objectivity in research. The values on which science is based – including honesty, fairness, collegiality, and openness – serve as guides to action in everyday life as well as in research” (NAS, NAE, COSEMPUP, 2009).

Not everyone agrees that scientists should engage in advocacy and activism. Some have argued that scientists should not take normative stances on any subject, and should therefore limit themselves to informing politicians, policymakers and the public about the ever-worsening state of life on Earth (Wells, 2018; Young, 2017). This argument assumes that science can ever exist outside the political arenas that frame how it is practised in the first place. In fact, the vast majority of fields that are now regarded as essential to the scientific community initially grew out of the conscious efforts to improve society and intervene in various crises. From genetic toxicology’s ties to the environmental movements of the 1960s and 70s (Frickel, 2004a) to “crisis disciplines” such as epidemiology and conservation biology, which aim to preserve the foundations supporting human and non-human life (Soulé, 1985; Susser and Bresnahan, 2001; Meine et al., 2006; Cox, 2007) – the struggle for social progress is often, if not always, intrinsically part of knowledge production (Choudry, 2020).

Scientists are, after all, citizens before scientists. All citizens in a society have a moral obligation to advocate for what they justifiably recognize to be right or good (Nelson and Vucetich, 2009). Thus, our role as citizens entails a responsibility to participate in the betterment of society to the best of our abilities. The fact that we are also scientists should not prevent us from being good citizens, or to abdicate our responsibilities to society. On the contrary, we enjoy the privileged position of having first-hand access to scientific knowledge, so our responsibility is even greater. Indeed, in a crisis situation, failure to advocate for particular positions can have devastating consequences, and even lead to preventable loss of life (Pietrucci and Ceccarelli, 2019; Benessia and De Marchi, 2017). Although many of us are working with non-permanent, short-term contracts – and often lack the rights of full citizens in the societies we live in – advocacy and activism can be a way to more deeply connect with those societies, while participating in global struggles that also affect our homelands (Frickel, 2004b).

Some scientists are, perhaps understandably, wary of damaging their credibility by getting involved in advocacy and activism. However, research indicates that this fear may be unfounded (Kotcher et al., 2017). In fact, the public expects scientists to be actively involved in critical issues, and to participate in policy advocacy, because of their scientific expertise (Cologna et al., 2021). Studies show that, when it comes to ecological activism, scientific objectivity is not inherently compromised by the political aspects of research and communication (Isopp, 2015). In the health sciences, for example, activism has been recommended as "sitting on a spectrum of possible health professional advocacy actions" (Bennett et al., 2020).

In contrast, the fear of being perceived as alarmist has seriously impeded accurate and effective scientific reporting by, for example, leading researchers to consistently underestimate the negative impacts of the climate and ecological crises (Brysse et al., 2013). It has also led many to be dispassionate in their communications, when a more passionate and emotional approach would have been more effective (Brosch, 2021). As Marcus and Oransky wrote in 2017: "Although the conduct of science demands honesty and rigour, nowhere is it written that researchers must remain silent when governments or other powerful players either misuse science or suppress findings in the service of harmful policies" (Marcus and Oransky, 2017).

## Expanding academic practices to include advocacy and activism

**Figure 1.**
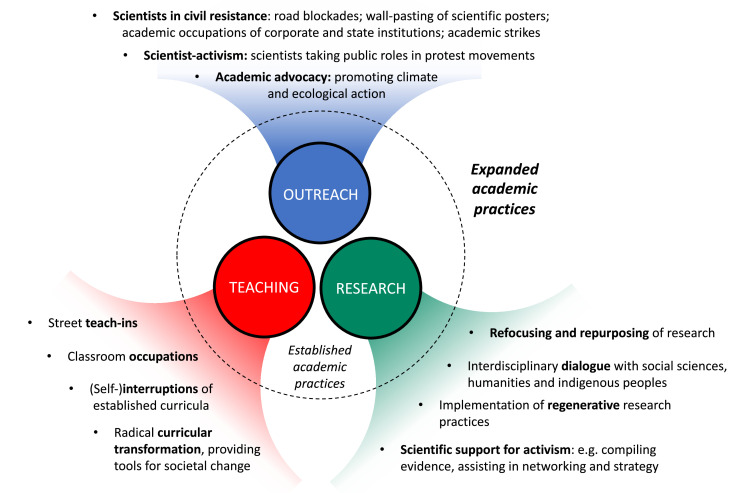
Examples of practices embracing advocacy and activism in academia. Scientists can expand the types of practices they carry out through their teaching, research and outreach, to better engage with the public and spur system change.

In this section we discuss how life scientists can include advocacy and activism in their work, in order to increase awareness of the climate and ecological crises, and to foster proactive responses to these crises. We will start with scientific outreach and then move to teaching and research (Figure 1).

Outreach is an essential aspect of being an academic, and connecting with the public can be done in many ways. In the biospheric emergency, however, outreach must go beyond conventional ways of making scientific knowledge available to the public. Instead, we argue, scientists must actively participate in movements that are openly engaging with the emergency, via effective forms of direct action that can garner mass media attention (Chenoweth et al., 2011; Chenoweth, 2020). Examples of such actions can include blockades of streets or bridges by scientists (Thompson, 2021), the occupation of corporate or government facilities (Chiarin, 2022), the pasting of pages from scientific papers and reports onto the walls of buildings (Gayle, 2022), and academic strikes (Ambjørseiet, 2022). As trusted communicators (Funk et al., 2020), scientists are also in a unique position to amplify voices of civil resistance that are currently under-powered. For instance, activists blocking the construction of an oil pipeline may be more likely to gain the attention and favour of the public if they are backed by biologists, ecologists or public health experts who can explain to journalists why new pipelines pose an existential risk to both nature and our civilization at large.

Similarly, our teaching practices can be adapted to embrace advocacy and activism, both inside and outside institutions of higher education. For example, scientists can organise street teach-ins on the biospheric emergency while blocking traffic (Nielsen and Thymark, 2021), interrupt our own course curricula with talks about the biospheric emergency (Steinberger, 2022), or even occupy university classrooms (Volkstimme, 2022), thereby putting pressure on institutions to seriously mobilise their resources. The emergency ultimately requires a radical transformation of academic curricula, to provide students not just with knowledge about the consequences of climate and ecological breakdown, but also with the social and political tools to address their causes (Steinberger, 2022).

Our research can also be expanded beyond business-as-usual – that is, beyond the generation of data, models, papers and reports that are largely targeted at other academics (Veríssimo et al., 2020). Instead of designing research programs for a world that will not exist anymore (IPCC, 2021), we can refocus and repurpose our research to develop tools that will help society to cope with the changes the biospheric emergency will cause. At the University of California San Diego, for example, the Climate Psychology and Action Lab is conducting research on socio-behavioural methods to help transition people away from climate skepticism or passivity, and into collective movements that confront fossil fuel extractivism and support renewable energies. Another recently launched initiative – Faculty for a Future – is helping academics transform their research, teaching, engagement, and institutions, so that they can, in turn, guide students and society into a more sustainable future. Along these lines, one of us (CJG) and colleagues recently argued that scientists can collaborate with activist groups, to help them collect evidence and improve strategies for movement-building and visibility (Gardner et al., 2021).

Scientists can also work to transition away from the unsustainable patterns of thinking in which academia has operated for decades, including a strong focus on conformism, competitiveness, opportunism and flexibility. These patterns of thinking are particularly dangerous in the biospheric emergency, as they have driven us to see technocratic approaches as the only solutions to humanity’s problems. Instead scientists must be clear about the kind of knowledge science is capable of producing – including its failures and limitations – without shutting down the concerns of the non-academically-trained public (Stengers, 2018).

Most critically, research in the biospheric emergency needs to involve local stakeholders across all stages of the process, including those who are most directly affected by climate and ecological breakdown (Funtowicz and Ravetz, 1993). It is vital for life scientists to listen to, collaborate with, and co-produce with social scientists, humanities scholars and indigenous communities: we must engage in meaningful dialogue with those who have been historically excluded or exploited by colonialism and neo-colonialism. For example, past and ongoing land-grabs, arrests and assassinations of Indigenous activists (Global Witness, 2018) have been key enablers of the climate and ecological crises, by silencing modes of thinking and understanding our world that questioned the socioeconomic norms violently imposed on other cultures (Salomon et al., 2018; Stein et al., 2021).

### Speaking clearly

Finally, if we are to provide society with an honest assessment of the problems at hand, we cannot shy away from naming the forces behind the climate and ecological crises. Statements that vaguely ascribe land degradation or species loss to “human activities” are often found in the academic literature: however, not all humans are equally responsible, nor are all human activities destructive of nature. Indeed, the people and countries most affected by climate change and ecological breakdown are also the least responsible for them (Gore, 2020; Oswald et al., 2020). Overly vague statements draw attention away from the specific social, economic and political systems that are driving the biospheric emergency. These systems include:

- **Extractivist capitalism**, which prioritises unfettered economic growth and environmental exploitation over long-term social and ecological well-being (Jackson, 2016; Hickel, 2020; Schmelzer et al., 2022)
- **Settler-state colonialism**, which uses state-sanctioned violence to displace, imprison or murder indigenous land care-takers (Whyte, 2018; Garnett et al., 2018; Stein et al., 2021; Global Witness, 2018)
- **Corporate capture of state institutions**, which makes elected officials unwilling to act in favour of the interests of the broader public (Lucas, 2021)
- **Citizen disenfranchisement**, which consists of barriers to public participation in democratic institutions, thereby perpetuating the inability of electoral politics to advance change (Smith, 2009; Gerwin, 2020; Gilens and Page, 2014).

Ultimately, we must be aware that scientific recommendations will be lost in the wind if they only take the form of polite appeals to dominant structures of power, particularly when those very same structures are the ones we are trying to transform. Scientists can facilitate change more effectively by talking to the public about why these structures prevent us from fully tackling the emergency, and how they can be reshaped through popular struggle (Steinberger, 2019; Steinberger, 2022).

## Scientists in rebellion

Many scientists are already engaging in some of these forms of activism (Thompson, 2021). In April 2022, for example, shortly after the publication of one of the IPCC reports, Scientist Rebellion mobilised over 1,500 scientists across 28 countries to highlight the dire global situation outlined by the report (IPCC, 2022b). Researchers of all scientific backgrounds risked arrest across all continents by participating in non-violent disruptive actions directed at governmental, scientific and corporate institutions deemed responsible for the climate and ecological crises (Harvey, 2022; Osborne, 2022). The IPCC report itself had been leaked by anonymous scientists almost a year in advance – another form of scientist activism – due to fears it would be watered down by politicians and corporate lobbyists before publication (Harvey, 2021). This fear was later shown to be founded (Bordera, 2022).

**Figure 2.**
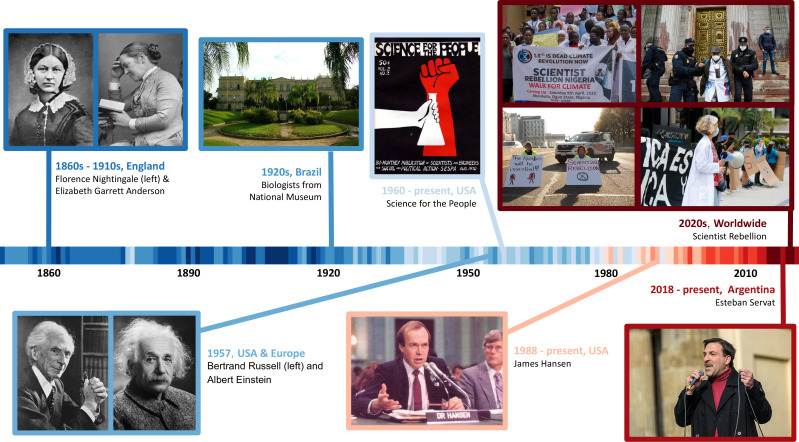
Scientists as activists through the ages. None of the tactics outlined in this article are new, and there is a long history of scientists engaging in activism. Examples include the work of statistician Florence Nightingale and physician Elizabeth Garrett Anderson in the women’s suffrage movement in the late nineteenth and early twentieth century; the activism of biologists from the National Museum in Brazil in favour of environmental protection in the 1920s; the Russell–Einstein manifesto of 1955 on the dangers on nuclear weapons; the Science for the People collective campaigning against war and for social justice in the United States in the late 1960s; the acts of civil resistance by climate scientist James Hansen to generate climate action; the work of biologist Esteban Servat building movements linking social justice and environmental abuse; and the Scientist Rebellion movement, which uses non-violent civil resistance in response to the climate and ecological crises. Image credits: Florence Nightingale: Henry Hering (CC0 1.0); Elizabeth Garrett Anderson: Walery (CC0 1.0); National Museum: Cyro A Silva (CC-BY 2.0); Bertrand Russell: Anefo (CC0 1.0); Albert Einstein: Orren Jack Turner (CC0 1.0); Science for the People (CC0 1.0); James Hansen: NASA (CC0 1.0); Esteban Servat: Stefan Müller (CC-BY 2.0); Scientist Rebellion Turtle Island (bottom left): Will Dickson (CC-BY 2.0); Scientist Rebellion Nigeria (top left): Obaloyin Timothy (CC-BY); Scientist Rebellion Spain (top right): Rodri Mínguez (CC0 1.0); Scientist Rebellion Panamá (bottom right): Viviano Romero, Renate Spooner (CC0 1.0); warming stripes: Ed Hawkins (CC-BY-SA).

Though regaining momentum today, scientist activism is not new (Figure 2). Its history is long, rich and diverse, testifying against the notion that it is unusual for researchers to get directly involved in societal problems (Moore, 2009). Prominent scientists like Albert Einstein, Bertrand Russell and Linus Pauling actively engaged in the debate on the development of nuclear weapons and the dangers of modern warfare (Russell et al., 1955; Pauling, 1958). American scholars and students participated in numerous teach-ins and strikes in the 1960s (Allen, 2019), as did the Science for the People collective in the late 1960s and early 1970s, in order to protest the US–Vietnam War. Prominent biologists, like Stephen Jay Gould and Richard Lewontin, were key figures in this anti-war mobilisation (Schmalzer et al., 2018). Likewise, activism has a proud tradition in the health sciences: scientist-campaigners like Florence Nightingale and Elizabeth Garrett Anderson contributed to major gains in social welfare rights for underprivileged groups, and in the fight for women’s right to vote (Launer, 2021).

Throughout the 20th and 21st centuries, well-known scientists have participated in activism specifically targeted against ecological deterioration. As early as the 1920s, activist biologists in Brazil were instrumental in influencing public policy to advance environmental protection policies (Duarte and Whitty, 2016). More recently, the climatologist James Hansen has repeatedly engaged in activism, ever since his historic testimony to the US Senate in 1988 to raise public awareness about climate change and its effects on the biosphere. This activism has often been in the form of civil resistance, leading to arrest (Bryner, 2013). Another example is the Argentine biologist Esteban Servat, who rose to global fame after publishing a secret government report on the disastrous effects of fracking in Mendoza, and was then forced into exile from his own country (Ketcham, 2019). He has also been responsible for co-organizing mass mobilisations across the world, highlighting links between social struggles in impoverished nations with environmental abuses by European and North American corporations (Ketcham, 2019; Monbiot, 2022).

## Moving forwards

This article has focused on the life sciences. However, we believe our recommendations are broadly applicable to all scientists and academics, regardless of their area of expertise. In times of emergency, the entire scientific community must act their part: we are among the most trusted members of society (Funk et al., 2020) and we cannot let our immense prestige, knowledge and privilege go to waste.

We set out to write this perspective because we are all currently participating in activism. We ask our colleagues, mentors, students and teachers to join us. Let us not turn ourselves into passive witnesses of mass death and extinction. We are life scientists after all – let’s stand up for life.

## Data Availability

N/A.
